# Metabolomic profiling of Marek’s disease virus infection in host cell based on untargeted LC-MS

**DOI:** 10.3389/fmicb.2023.1270762

**Published:** 2023-11-09

**Authors:** Qingsen Wang, Bin Shi, Guifu Yang, Xueying Zhu, Hongxia Shao, Kun Qian, Jianqiang Ye, Aijian Qin

**Affiliations:** ^1^The International Joint Laboratory for Cooperation in Agriculture and Agricultural Product Safety, Ministry of Education, Yangzhou University, Yangzhou, Jiangsu, China; ^2^Jiangsu Co-innovation Center for Prevention and Control of Important Animal Infectious Diseases and Zoonoses, Yangzhou, Jiangsu, China; ^3^Jiangsu Key Laboratory of Zoonosis, Yangzhou, Jiangsu, China

**Keywords:** Marek’s disease virus, metabolites, amino acid, the TCA cycle, CEFs, LC-MS

## Abstract

Marek’s disease (MD) caused by Marek’s disease virus (MDV), poses a serious threat to the poultry industry by inducing neurological disease and malignant lymphoma in infected chickens. However, the underlying mechanisms how MDV disrupts host cells and causes damage still remain elusive. Recently, the application of metabolomics has shown great potential for uncovering the complex mechanisms during virus-host interactions. In this study, chicken embryo fibroblasts (CEFs) infected with MDV were subjected to ultrahigh-performance liquid chromatography-quadrupole time-of-flight tandem mass spectrometry (UHPLC-QTOF-MS) and multivariate statistical analysis. The results showed that 261 metabolites were significantly altered upon MDV infection, with most changes occurring in amino acid metabolism, energy metabolism, nucleotide metabolism, and lipid metabolism. Notably, MDV infection induces an up-regulation of amino acids in host cells during the early stages of infection to provide the energy and intermediary metabolites necessary for efficient multiplication of its own replication. Taken together, these data not only hold promise in identifying the biochemical molecules utilized by MDV replication in host cells, but also provides a new insight into understanding MDV-host interactions.

## Introduction

Marek’s disease (MD), caused by Marek’s disease virus (MDV), is a highly contagious tumor disease in chickens that results in malignant lymphomas and nerve damage (McPherson and Delany, 2016). MDV induced diseases have undergone significant changes since their discovery, and can currently be classified into mild (m), virulent (v) very virulent (vv), and very virulent plus (vv+) based on their pathological properties (Dunn and Gimeno, 2013). GA belongs to the V strain and can cause lymphoma, with a mortality rate of up to 23% (Zhang et al., 2016). Previous studies *in vitro* and *in vivo* have elaborated on the pathogenesis with the time course of MDV infection from various perspectives (Chien et al., 2012; Hu et al., 2012, 2015; Pauker et al., 2019; Jin et al., 2020; Zheng et al., 2023). During the lytic phase of MDV infection, transcriptomic data suggests that there are more differentially expressed genes in splenic lymphocytes than in bursal lymphocytes, indicating an immune response to MDV infection in splenic lymphocytes (Jin et al., 2020); during the latency phase, the inhibition of apoptosis in host cells results in sustained viral infection and spread of the virus during the latent period (Zheng et al., 2023). In order to survive and replicate in host cells, viruses hijack the host’s energy and metabolic products for their own synthesis of virus genomes and proteins. MDV has been shown to hijack glycolysis and protein synthesis to enhance its replication in host cells. MDV infection can induce the enhancement of arginase activity in macrophages. This activation may be the result of the direct or indirect effects of the virus on macrophages, affecting the balance of cellular metabolism. Activation of arginase leads to changes in the metabolism of various amino acids, including arginine. These changes play a key role in promoting tumorigenesis (Djeraba et al., 2002). In addition, MDV infection can regulate the metabolic reprogramming of host cell glycolysis. MDV infection increases the activation of mitochondrial fatty acid β-oxidation, which can increase the oxygen consumption rate (OCR) in MDV-infected cells, ultimately leading to an increase in glycolysis to promote virus replication (Boodhoo et al., 2020b).

Although these studies have expanded our understanding of MDV, the metabolic requirements of MDV still remain elusive. This is evident in the modification of metabolic signaling pathways in host cells during the infection process (Fabricant et al., 1981; Hajjar et al., 1986), which may have a significant impact on the pathogenesis of MDV. Recently, researchers have started using metabolomics techniques to investigate the pathogenic mechanisms of cancer and viruses from a host metabolic perspective (Santana-Codina et al., 2018; Wang et al., 2019; Pareek et al., 2020; Ariav et al., 2021). The field of metabolomics has emerged as a key discipline in the wake of genomics and proteomics. Metabolomics study low-molecular-weight metabolites (MW < 1 KD) involved in various metabolic pathways, such as sugars, lipids, amino acids, and vitamins. This approach can detect alterations in metabolic responses of cells or tissues that result from external stimuli or genetic modifications (Johnson et al., 2016; Parveen et al., 2019). At present, metabolomics holds significant promise in unveiling intricate virus-host interactions. For example, heightened levels of 7-dehydrocholesterol have been identified as an indicator of cholesterol metabolism dysfunction in host cells infected with hepatitis B virus (HBV) (Rodgers et al., 2009). The impact of HSV-1 on cellular metabolic homeostasis in both quiescent and actively growing cells has been demonstrated, with the virus being shown to induce changes in several metabolic pathways, including glycolysis, the tricarboxylic acid (TCA) cycle, and pyrimidine biosynthesis (Vastag et al., 2011). It is conceivable that the host metabolic pathways may be interfered during viral replication. Despite these interesting observations, it is not clear that the mechanism of MDV regulates the small molecule metabolites production to facilitate macromolecular synthesis. Further studies are necessary to uncover the exact mechanisms how MDV affects small molecule metabolite production and macromolecular synthesis, thereby providing a deeper insight into this fascinating biological phenomenon.

In this study, we employed an untargeted metabolic approach based on ultrahigh-performance liquid chromatography-quadrupole time-of-flight tandem mass spectrometry (UHPLC-QTOF-MS) to comprehensively detect and analyze a wealth of metabolites. This analytical method allowed us to perform a completed investigation of the metabolic landscape, providing valuable insights into the metabolic changes associated with MDV infection. Our findings indicated that a number of metabolites and metabolic pathways undergo pronounced alterations during MDV infection. These alterations suggest that they may enhance the process of viral replication, thereby providing a deeper understanding of the host’s response to MDV infection and offering new perspectives for the control of MD.

## Materials and methods

### Viruses and cells

MDV strain GA was preserved in our laboratory and belongs to virulent (v) (Lee et al., 2000). Nine-day-old specific-pathogen-free embryonated chicken embryos (purchased from Lihua) were used to generate chicken embryo fibroblasts (CEFs). As described in our previous report (Zai et al., 2022), CEFs were cultured with M199 medium (Gibco), supplemented with 5% fetal bovine serum (FBS; Lonsera), 2 mM glutamine, 100 U/mL penicillin, and 100 mg/mL streptomycin (HAKATA). The cells were seeded into the culture flasks (SORFA) and placed at 37°C in a humidified atmosphere with 5% CO_2_.

### Virus infection

For viral infection, cells were infected with MDVs at a multiplicity of infection (MOI) of 0.01 in M199 for 6 h at 37°C. After adsorption, the supernatant was removed to eliminate viral particles that were not internalized, and the remaining solution was cultured in fresh M199 with 1% fetal bovine serum for further analysis. Samples from CEFs were collected at 0, 24 and 36 h post infection (hpi).

### Western blotting

Cell extracts were collected using lysis buffer (GenStar) with added protease inhibitor (TransGen Biotech) as described report (Wang et al., 2021), then centrifuged at 12,000 × g for 15 min at 4°C to obtain supernatants. The lysates were mixed with SDS-PAGE Loading Buffer (CWBIO) and heated at 95°C for 5 min. Samples were separated using SDS-PAGE (MeilunBio) and transferred onto Immobilon-NC membranes (Absin) at 250 mA for 120 min. The prestained dual color protein molecular weight marker was purchased from LABLEAD. The membrane was closed with 5% skim milk in Tris-buffered saline-Tween (TBST) at room temperature for 2 h prior to incubation with primary antibodies mAb anti-gB BA4 (Zhuang et al., 2015) and β-actin (Bioss) at 4°C overnight, and goat anti-mouse HRP-conjugated secondary antibodies (1 h; Sangon). The protein band was developed with chemiluminescence detection reagents (NCM Biotech) and visualized using photographed with an ultrasensitive chemiluminescence detector (ProteinSimple). Use a colorful protein marker (MeilunBio) to estimate the size of the protein bands.

### Indirect immunofluorescence analysis

For infected cells, they were fixed with acetone ethanol (3:2) for 15 min at room temperature to fix cells, and then the cells were washed 3 times with phosphate-buffered saline (PBS). The fixed cells were incubated with mAb anti-gB BA4 (Zhuang et al., 2015) at 4°C overnight before incubating with FITC-labelled goat anti-mouse secondary antibody (KeyGEN) incubated in a 37°C water bath for 30 min. These pictures were captured with an OLYMPUS fluorescence microscope.

### Metabolomics

Cells infected with MDV-GA strain were collected at 0 h, 24 h, and 36 h, with 4 replicate samples of 1.5 × 10^7^ cells per group, centrifuged at 1000 rpm for 2 min, and cell precipitates were collected and frozen at −80°C refrigerator. Samples were sent to Shanghai Applied Protein Technology for nontargeted metabolomics analysis. The samples were thawed at 4°C and mixed with 1 mL of cold methanol/acetonitrile/H2O (2:2:1, v/v/v). The homogenate was sonicated for 30 min once and centrifuged for 20 min (14,000 g, 4°C). The supernatant was dried in a vacuum centrifuge and re-dissolved in 100 μL acetonitrile/water (1:1, v/v) solvent. UHPLC (1,290 Infinity LC, Agilent Technologies) coupled to a quadrupole time-of-flight (AB Sciex TripleTOF 6,600) was used for LC-MS analysis. For HILIC separation, a 2.1 mm × 100 mm ACQUIY UPLC BEH 1.7 μm column (waters, Ireland) was used with mobile phase containing A = 25 mM ammonium acetate and 25 mM ammonium hydroxide in water and B = acetonitrile. The gradient was 95% B for 0.5 min and was linearly reduced to 65% in 7 min, and then was reduced to 40% in 0.1 min and kept for 1 min, and then increased to 95% in 0.1 min with a 3 min re-equilibration period. For RPLC separation, a 2.1 mm × 100 mm ACQUIY UPLC HSS T3 1.8 μm column (waters, Ireland) was used with mobile phase containing A = water with 0.1% formic acid and B = acetonitrile with 0.1% formic acid (ESI positive) or 0.5 mM ammonium fluoride in water (ESI negative). The gradient was 1% B for 1.5 min and was linearly increased to 99% in 11.5 min and kept for 3.5 min. Then it was reduced to 1% in 0.1 min and a 3.4 min of re-equilibration period was employed. The gradients were at a flow rate of 0.3 mL/min, and the column temperatures were kept constant at 25°C. QC samples were inserted into the sample queue to monitor system stability and experimental data reliability. A 2 μL sample was injected. ESI source conditions were set with Gas1 and Gas2 at 60 and CUR at 30, source temp at 600°C, and ISVF at ±5,500 V. MS only acquisition was set for m/z 60–1,000 Da with a scan time of 0.20 s/spectra. Auto MS/MS acquisition was set for m/z 25–1,000 Da with a scan time of 0.05 s/spectra using IDA with high sensitivity mode selected. Parameters were set with CE fixed at 35 V ± 15 eV, DP at 60 V (+) and − 60 V (−), excluding isotopes within 4 Da, and monitoring 10 candidate ions per cycle.

### Quantitative real-time PCR

According to the manufacturer’s instructions, total RNA was isolated from cells in 6 well plates (NEST Biotech) using FastPure Cell/Tissue Total RNA Isolation Kit V2 (RC112 Vazyme Biotech). Single-strand cDNA was generated from total RNA and reverse transcriptase (Cat#11143 Yeasen). The SYBR q-PCR SuperMix (Novoprotein, Shanghai, China) was used for quantitative real-time PCR analysis. Gene expression levels were analyzed using the 2^−ΔΔCT^ method, using the GAPDH gene as internal control. All analytical procedures were repeated for three biological replicates (Wang et al., 2021). Primers are listed in Table 1.

**Table 1:** Primers used in this study.

Gene ID	Gene name	Sequence of primers (5′–3′)
374193	GAPDH-F	AGGGTGGTGCTAAGCGTGTTA
	GAPDH-R	TCTCATGGTTGACACCCATCA
417185	ASS1-F	GGCTACACTGTCATCGCCTT
	ASS1-R	GGCCAGATGAAGTCCTCCAC
373916	Aco1-F	AGATTGTTGAGCCACTGGAT
	Aco1-R	TGCTTCCAAGAGGACTCGAA
374009	Aco2-F	ATGGGTGTCAAATGGGCAGT
	Aco2-R	TCTGCTGGATCCGCAAAAGT
431056	IDH2-F	AAGATGGTCTTCACGCCGAA
	IDH2-R	GTGTTGTACATGCCCATGCC
426429	OGDH-F	GCTCGATCCTCTCGGCATTA
	OGDH-R	CAGCCCGTAAAATCCGACG
423612	GLUD2-F	GCCATCAGAAGGTCGTGACA
	GLUD2-R	AGTTTCTGGGAGGGACTGGT
396,261	GOT1-F	CGCTGCTGTTATGAAGCGAC
	GOT1-R	ACAAAGTATCGCACAGCCCA
416642	ABAT-F	GGAACGATGTGCAAAGACACC
	ABAT-R	GGTGAGCCCGAAACGTTCAT
395395	GAD2-F	TTCCCAGACTGGTTGCCTTC
	GAD2-R	CGCATCCAATCAGAATCACACT
396498	ASL1-F	GCCGGGTGGACTTGATAGTA
	ASL1-R	CTCCACACTGTTGACAACGC
AF147806.2	MDV-Meq-F	GTCCCCCCTCGATCTTTCTC
	MDV-Meq-R	CGTCTGCTTCCTGCGTCTTC
AF147806.2	MDV-gB-F	ACCCCATTCGGTGGCTTTTC
	MDV-gB-R	GCGTCCAGTTGTCTGAGG

### Statistical analysis

The initial MS data files were transformed into MzXML files with ProteoWizard MSConvert and then analyzed using the freely available XCMS software. Specific parameters were utilized for the peak detection, including centWave m/z at 25 ppm, peakwidth ranging from 10 to 60 units, and prefilter between 10 and 100 units. Peak grouping was then performed with the parameters bw at 5, mzwid at 0.025, and minfrac at 0.5. Variables were selected based on the criterion that they had more than 50% of nonzero measurements in one group of the extracted ion features. Finally, metabolites were identified by comparing their MS/MS spectra to an in-house database of authentic standards. Normalized to total peak intensity.

The processed data were normalized to total peak intensity and uploaded to SIMCA-P (version 16.1, Umetrics, Umea, Sweden) for multivariate data analysis, including Pareto-scaled principal component analysis (PCA) and orthogonal partial least-squares discriminant analysis (OPLS-DA). The model was evaluated for robustness using 7-fold cross-validation and response permutation testing. Variable importance in the projection (VIP) value was calculated for each variable in the OPLS-DA model to determine its contribution to classification. Metabolites with a VIP score greater than 1 were analyzed using a Student’s *t*-test at the univariate level to assess their statistical significance (*p* < 0.05).

Metabolite annotation is conducted by referencing the KEGG Compound database, with subsequent mapping to the KEGG Pathway database. Significant enrichment pathways are discerned through hypergeometric tests, offering a comprehensive contextualization of the identified metabolites within broader biological pathways. The KEGG pathway analyses were performed using the cloud.genepioneer.com.

## Results

### Replication of MDV in CEFs

**Figure 1:**
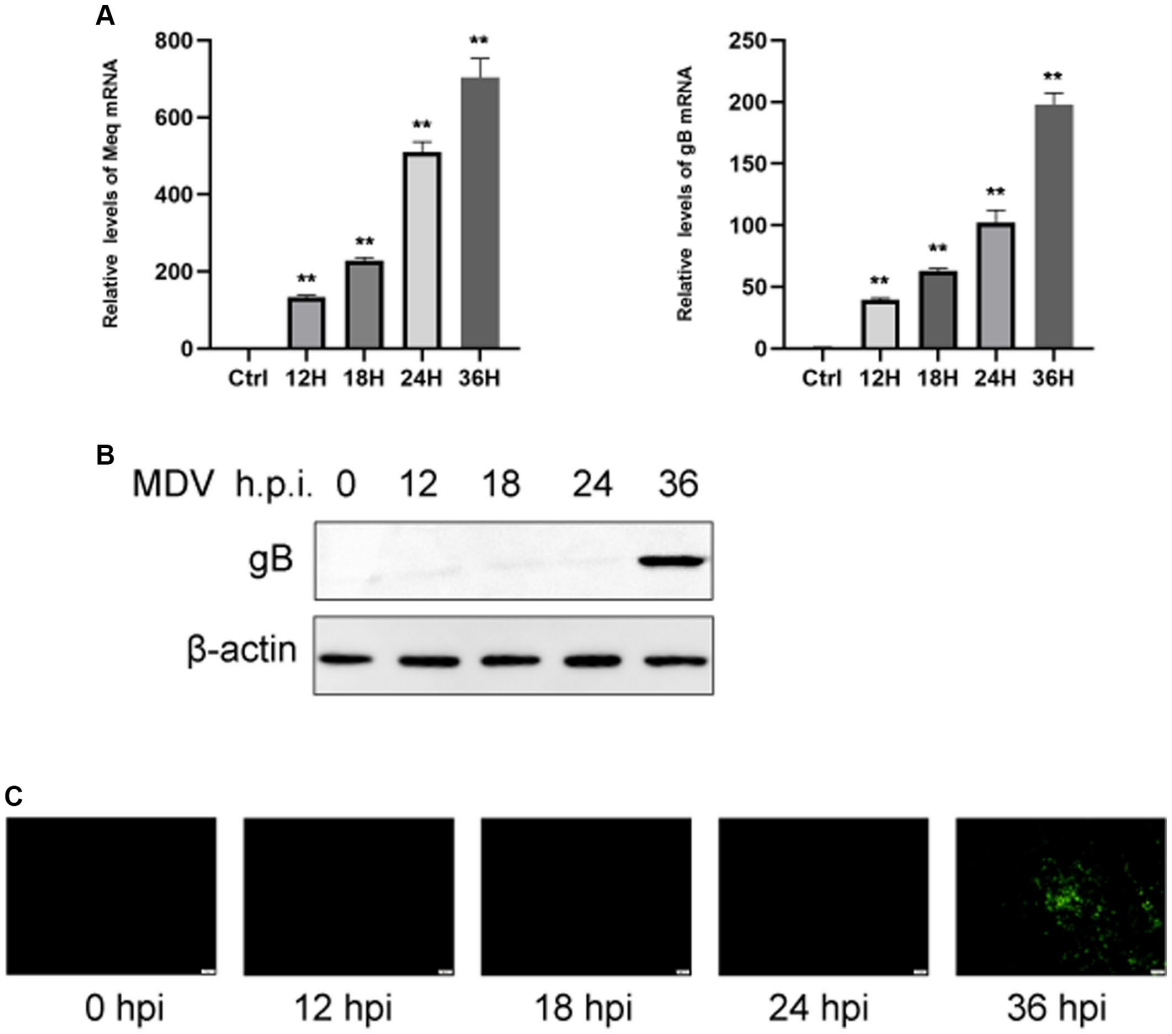
CEFs were infected with MDV at different time points. **(A)** q-PCR was used to find viral Meq and gB gene expression. Expression of viral gB protein was detected by Western blotting **(B)**. **(C)** Post-MDV infection immunofluorescence staining of CEFs. Cells with a MOI of 0.01 were distributed throughout a 24-well plate. FITC-conjugated antibody was used to examine the MDV gB protein. On the right was a control that wasn’t infected. Scale bar, 50 μm. The data represent the mean ± S.D. **p < 0.01.

To determine the replication ability of MDV in CEFs, cells were infected with the MDV-GA strain at a MOI of 0.01. Our results demonstrated that the replication rates of the virus increased over time. By using q-PCR analysis, we observed that two viral genes, Meq and gB, had an obvious increase in transcriptional levels at 24 and 36 hpi (Figure 1A). Furthermore, we assessed the levels of the viral protein gB using Western blot and IFA assays. It can be observed that the gB protein level gradually increased over time (Figure 1B). IFA imaging confirmed the presence of the virus (Figure 1C). Therefore, cells were collected at 0, 24, and 36 hpi for the subsequent analysis through metabolomics.

### Metabolic analysis in response to MDV infection by LC-MS

**Figure 2:**
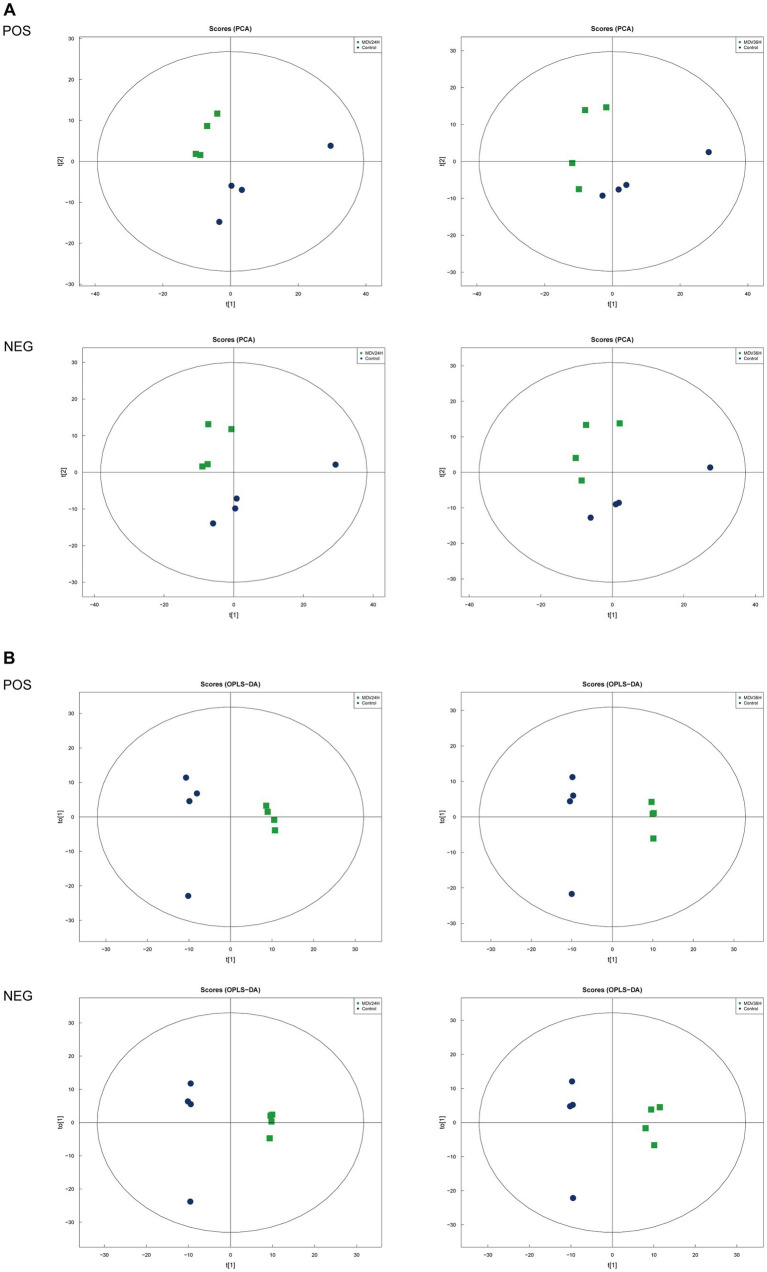
Score scatter plots of PCA and OPLS-DA of MDV-infected and uninfected cells. **(A,B)** Score scatter plot of the PCA and OPLS-DA model for the different infection groups versus ctrl group. **(A)** Score scatter plots of PCA and OPLS-DA of MDV-infected and uninfected cells. **(A,B)** Score scatter plot of the PCA and OPLS-DA model for the different infection groups versus ctrl group. **(A)** PCA diagrams correspond to different groups. T [1] represents the first principal component, t [2] represents the second principal component; each point in the figure represents a sample, and samples from the same group are represented by the same color and **(B)** in the OPLS-DA model, t [1] represents principal component 1, to [1] represents principal component 2, and the ellipse represents the 95% confidence interval. The dots of the same color represent various biological replicates within a group, and the distribution status of the dots reflects the degree of difference between and within groups.

To determine whether host cell metabolism is altered during MDV replication, we gathered the positive (POS) and negative (NEG) ion mode metabolomics data and used the PCA model to compare the differences between the mock group and infected groups. The PCA scatter plot results showed that all samples were within the 95% confidence interval and that the mock group and infected groups were separated into distinct regions (Figure 2A).

To provide a clearer representation of the metabolic changes occurring at 24 and 36 hpi, we performed an OPLS-DA supervised multidimensional statistical analysis. Figure 2B presents the results of this analysis, displaying the OPLS-DA data for both the mock group and the infected groups. These evaluation parameters of the OPLS-DA model (Table 2) indicate a trustworthy model, as the Q^2^ values are greater than 0.5. These results suggest that the mock group and infected groups are easily distinguishable, and the OPLS-DA models have been trusted. The host cell metabolism was clearly altered during MDV replication.

**Table 2:** OPLS-DA model for MDV infection group versus ctrl group.

		POS			NEG		
Title	Type	R^2^X (*cum*)	R^2^Y (*cum*)	Q^2^ (*cum*)	R^2^X (*cum*)	R^2^Y (*cum*)	Q^2^ (*cum*)
MDV24Hours control	OPLS-DA	0.57	0.99	0.829	0.607	0.999	0.777
MDV36Hours control	OPLS-DA	0.676	0.999	0.843	0.464	0.992	0.706

### Differential metabolite analysis in CEF infected with/without MDV

To search for differentially metabolites during MDV infection, we evaluated all metabolites found in positive and negative ion mode (including unidentified metabolites) based on univariate analysis. We represented the differential metabolites, which showed Changes of >1.5-fold or < 0.67-fold and had a *p*-value from Student’s *t*-test of less than 0.05 (Supplementary Figure S1).

**Figure 3:**
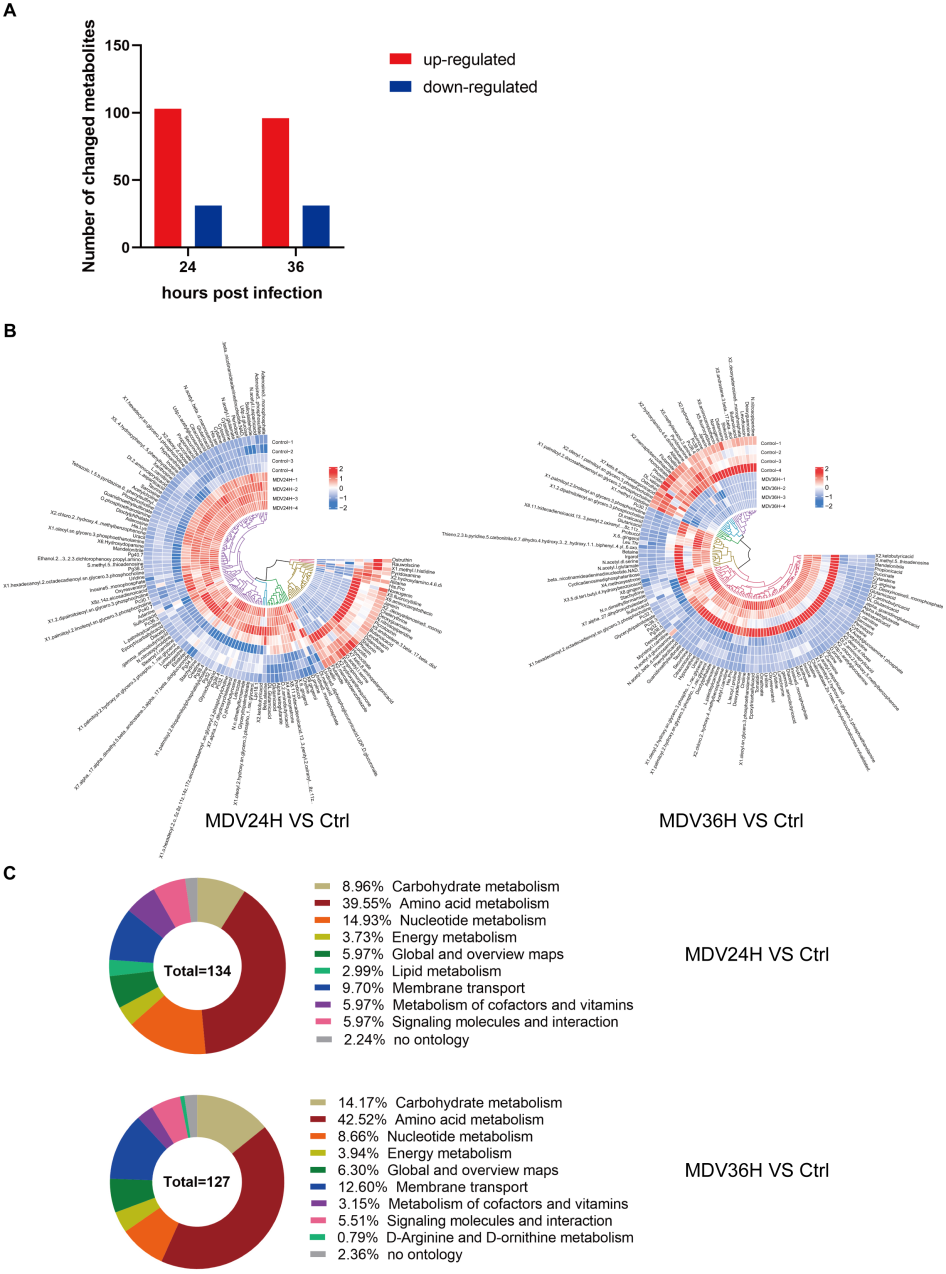
**(A)** Using VI *p* value (>1) and *p* value <0.05 as screening criteria, differentially significant metabolites that are up-regulated and down-regulated in MDV-infected cells are displayed in red and blue, respectively. **(B)** Heat map of the examination of different metabolites using hierarchical clustering. One sample is represented by each column, and one different metabolite is represented by each row. Upregulation in red; downregulation in blue. **(C)** Pie graph showing the distribution of metabolite superclasses. The metabolomics data included annotations for 134 perturbed metabolites (24 hpi) and 127 perturbed metabolites (36 hpi). Each section is colored to represent a certain class of metabolites.

The OPLS-DA model’s VIP was able to quantify the intensity and interpretability of the patterns of each metabolite on the samples’ ability to distinguish between various taxonomic groups and to mine the biologically significant differentially metabolite molecules. The VI *p* value (>1) of the OPLS-DA model and the *p* value <0.05 were used as the criteria for significant differential metabolite screening. We identified 261 significantly different metabolites in MDV-infected cells, including 199 metabolites that were upregulated and 62 that were downregulated (Figure 3A). A heatmap analysis showed that glutamic acid, deoxyguanosine, adenosine, and alpha-ketoglutarate were all significantly upregulated after infection (Figure 3B). At 24 and 36 hpi, our data revealed that the fraction of metabolites associated with amino acid metabolism was greatest, accounting for 39.55% and 42.52% of all metabolites, respectively (Figure 3C). This evidence demonstrates that MDV infection can significantly alter the metabolism of CEFs.

### Metabolic pathway analysis of CEF cells infected with MDV

**Figure 4:**
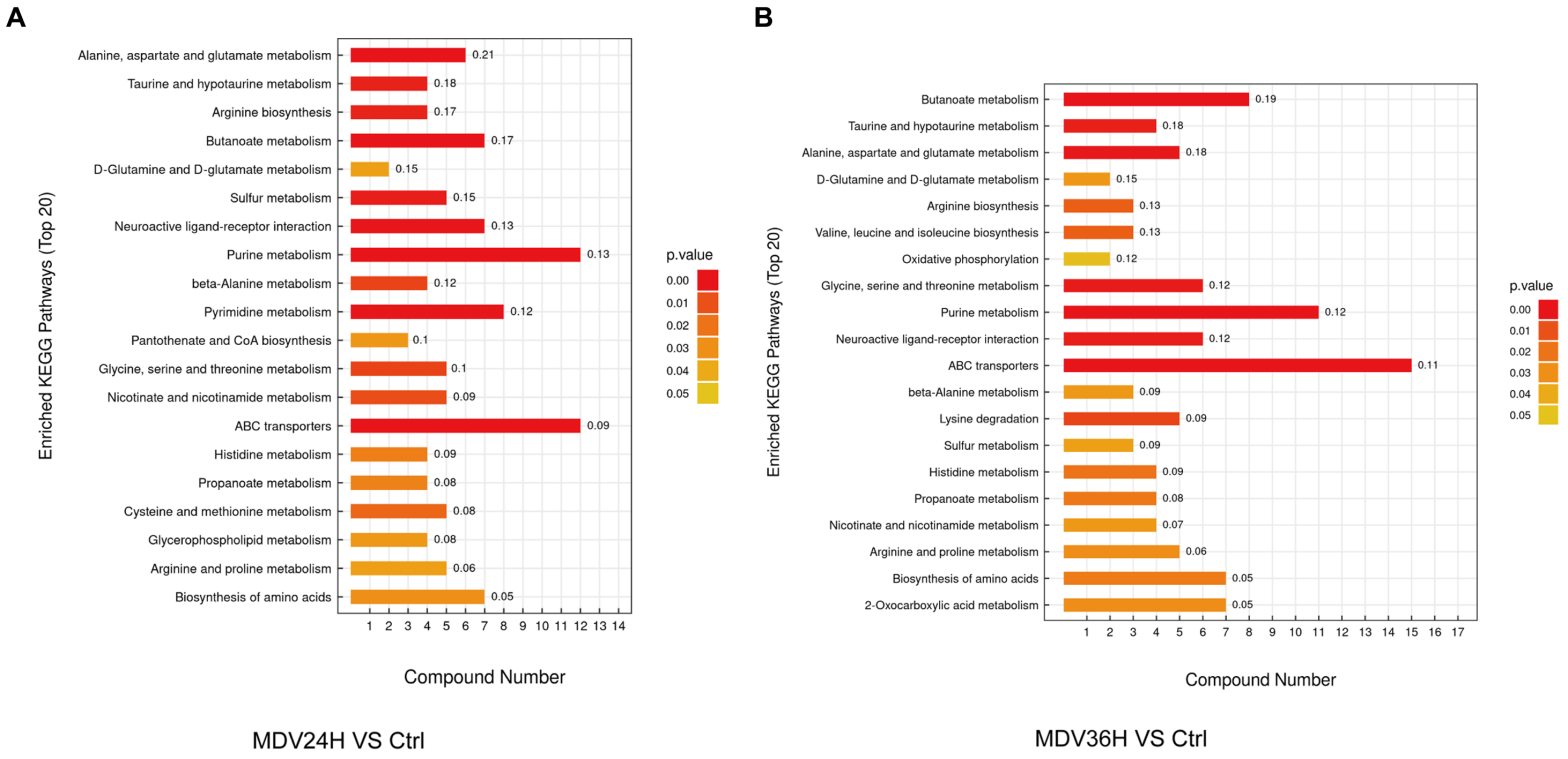
In the KEGG enrichment pathway diagram, the vertical axis represents different KEGG metabolic pathways, and the horizontal axis indicates the number of significantly differential metabolites contained within each pathway. Color represents the *p*-value of the enrichment analysis, with deeper colors indicating smaller *p*-values and more significant degrees of enrichment. The number on the column represents the “enrichment factor,” which represents the ratio of differentially expressed metabolites in the pathway to the total number of annotated metabolites in that pathway. Each diagram selects the top 20 most significant pathways based on the *p*-value. **(A)** MDV24H *VS* Ctrl; **(B)** MDV36H *VS* Ctrl.

We analyzed the biological implications of the changes in metabolites by the differential metabolites with the KEGG Metabolome Database, which specializes in metabolic pathway identification and enrichment. 20 most significant metabolic pathways, as determined by the *p* value, were presented the results in bar plots (Figure 4). The findings revealed that a wealth of metabolites was significantly altered after MDV infection and were linked to several key metabolic pathways, including alanine, aspartate, and glutamate metabolism, purine metabolism, butanoate metabolism, amino acid biosynthesis, aminoacyl-tRNA biosynthesis, and ABC transporters. These findings suggest that MDV infection has a pronounced impact on the host cell metabolism.

### Analysis of metabolite network in amino acid biosynthesis pathway during MDV infection

**Figure 5:**
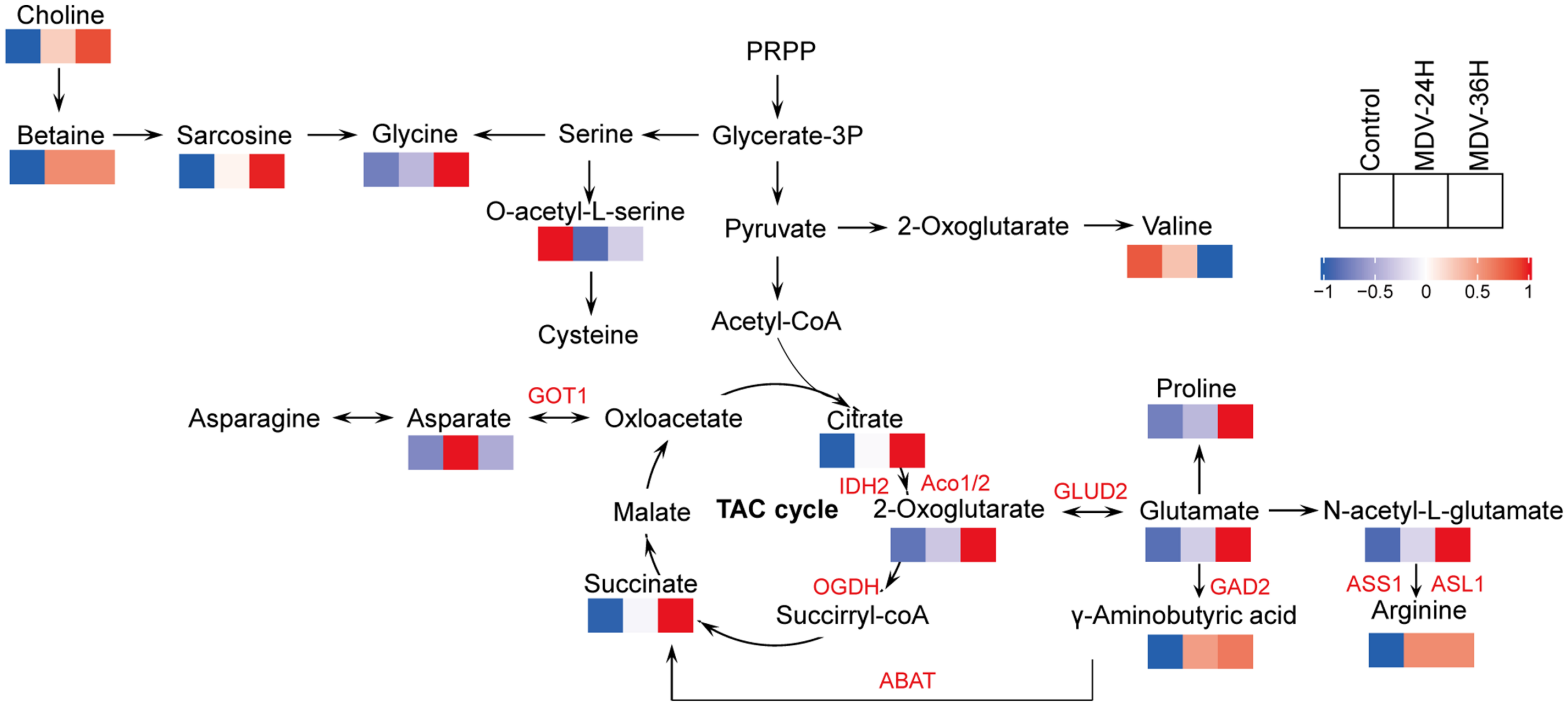
Overview of the modified metabolic pathways in MDV-infected CEFs using a schematic. The original relative content of differential metabolites is standardized by row, and different colors are filled with different values obtained after standardization of different relative contents. Blue: downregulated; red: upregulated.

An increase in metabolites related to glycine production was observed, including choline, betaine, and sarcosine, at 24 and 36 hpi with the MDV-GA strain. At the same time, there was significantly increase of other amino acids, such as glutamate, proline, N-acetyl-L-glutamate, and arginine, which is consistent with the replication of the virus (Figure 5). We also observed changes in the TCA cycle and amino acid synthesis, as demonstrated by the increased levels of α-ketoglutarate, citric acid, succinic acid, and γ-aminobutyric acid in infected cells at 24 and 36 hpi. These results suggest that the metabolism of the host cells is altered in response to MDV infection.

### Validation of candidate genes associated with amino acid metabolism during MDV infection

**Figure 6:**
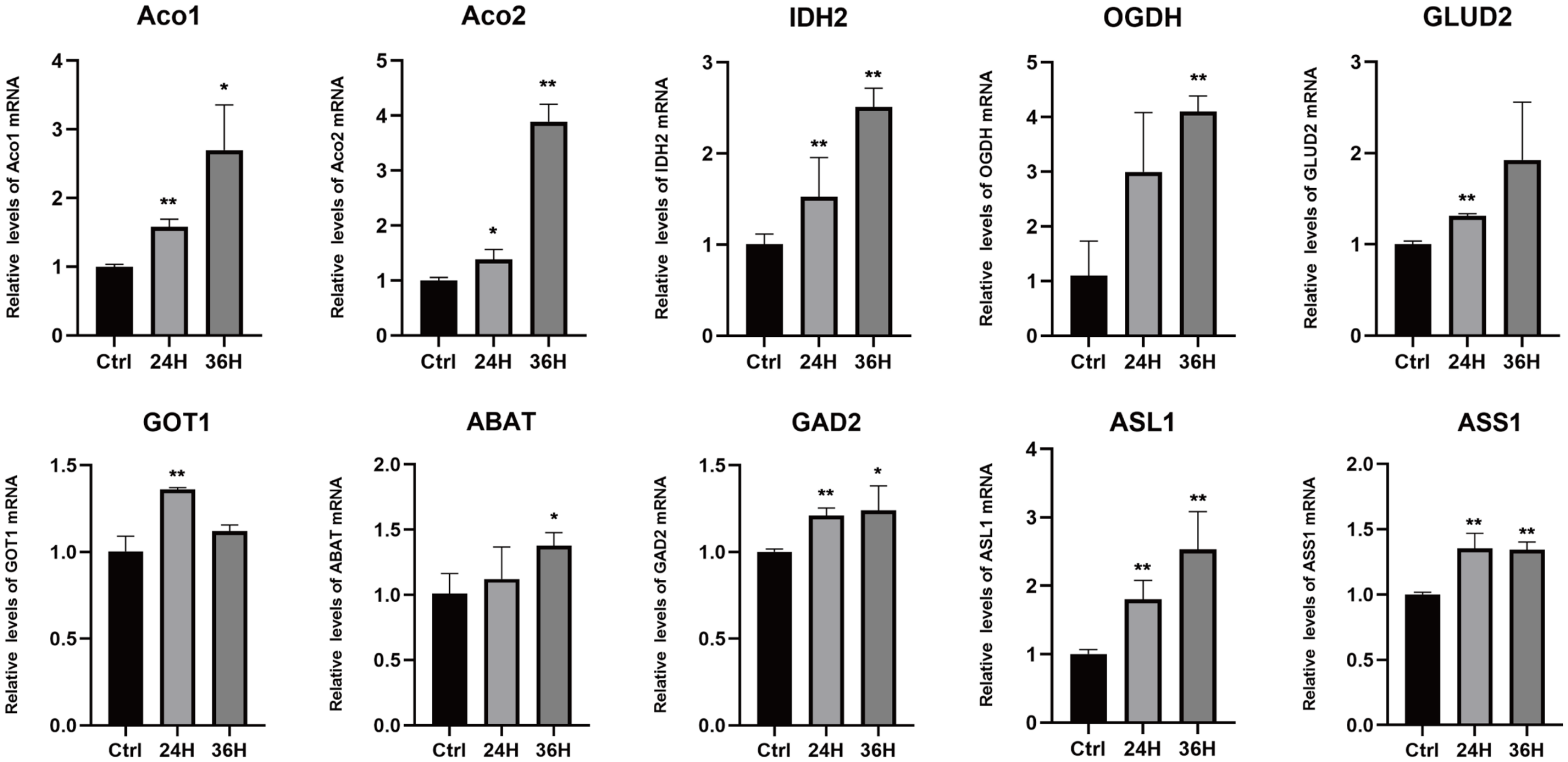
The mRNA levels of enzymes involved in the metabolism of amino acids following MDV infections. The mRNA levels of enzymes involved in the metabolism of amino acids following MDV infections. Total mRNA was isolated from mock- and MDV infected CEFs at the indicated time points, and q-PCR were performed with ten genes that regulate amino acids enzymes specific primers. Results were normalized to the level of GAPDH expression and are expressed as the mean fold change in regulate amino acids enzymes mRNA expression compared to that in Ctrl cells ± SD.

To explore the role of amino acids in MDV replication, we conducted a q-PCR analysis to investigate the relationship between alterations in amino acid metabolism and the expression of key amino acid regulatory enzymes (Figure 6), including aconitase 1/2 (Aco1/2), isocitrate dehydrogenase 1 (IDH2), oxoglutarate dehydrogenase (OGDH), glutamate dehydrogenase 2 (GLUD2), glutamic-oxaloacetic transaminase 1 (GOT1), 4-aminobutyrate aminotransferase (ABAT), glutamate decarboxylase 2 (GAD2), argininosuccinate lyase 1 (ASL1), and argininosuccinate synthase1 (ASS1). Consistent with metabonomic data, these metabolic changes are related to the increase of the expression of key enzymes that regulate amino acid metabolism. Our results showed that the mRNA levels of Aco1, Aco2, IDH2, OGDH, GLUD2, ABAT, and ASL1 were increased following MDV infection, indicating these genes may be functionally linked to viral replication. These results suggest that the infection with MDV had a significant effect on the metabolic pathways associated with amino acids.

## Discussion

MDV undergoes four overlapping infection stages that have critical consequences contributing to viral persistence and pathogenesis in the host: early cytolytic, latent, late cytolytic, and transformation (Bertzbach et al., 2020). Firstly, in the stage of cell lysis, viruses replicate in cells. This stage provides energy and raw materials for virus replication through the metabolism of host cells. However, the relationship between virus infection and host metabolism is complex and diverse, making it difficult to clarify the specific changes that occur in host metabolism at this time. Nitish’s research suggests that glycoprotein B of MDV is associated with LAMP-1. The gene silencing of LAMP-1 reduces the replication and transmission of MDV, highlighting the importance of LAMP-1 as a gB transporter protein. The results indicate that MDV hijacks cell cholesterol biosynthesis and cholesterol transport to promote intercellular transmission through LAMP-1 dependent mechanisms (Boodhoo et al., 2020a). The advances in metabolomics have now made it possible to gain new insights into the intricate metabolic networks within host cells that allow for the proliferation of viruses (Munger et al., 2008). With the advantage of metabolomics, researchers are now able to unravel the complex web of metabolic pathways underlying a wide range of physiological and pathological processes (Naviaux et al., 2016; Wishart, 2019; Fu et al., 2023). In recent years, the field of metabolomics has provided new clues on how viruses interact with host cells, including how infections impact cellular metabolism. For instance, a study on the pseudorabies virus (PRV) revealed that variant strain (HNX) consumes more photoshatidylglycerol (PG), but also promotes PG synthesis compared to PRV vaccine strain (Bartha K61). PRV-induced lipid metabolic pathways alternations may link with viral assembly and egress. These findings illustrate the complex relationship between virus infections and cellular metabolism (Yao et al., 2021). In order to investigate how MDV promotes its replication in host cells by reprogramming metabolic features, CEF cells were selected as the viral infection model, which is commonly used to study the *in vitro* growth of MDV. In order to reveal the basic metabolic characteristics and tumorigenic mechanism of MDV infected cells, CEF cells were infected with MDV-GA strain and analyzed them using LC-MS technology. According to reports, this virulent strain belongs to virulent strain and that can cause malignant tumors upon *in vivo* infection (Zhang et al., 2016). The metabolic changes that occur during the MDV infection process were systematically investigated through dynamic metabonomic analysis. The OPLS-DA and PAC studies, represented in heat maps, revealed a stark contrast in metabolic parameters between the MDV-infected group and the simulated control group. It indicated that the initial metabolic balance of CEF cells is disrupted by MDV infection, resulting in notable alterations in amino acid metabolism and the TCA cycle. These changes trigger a metabolic re-programming response and result in an increase in metabolites, reflect the virus’s need for resources during the replication process (Figure 5). This research sheds new light on the intricate interplay between MDV and host metabolism.

Amino acids, the fundamental building blocks of life, are not only required for maintaining cellular and viral functions, but they also play a central role in the formation of cellular and viral proteins. For example, during the replication of Gallid alphaherpesvirus 1 (ILTV), glutamine plays a pivotal part in the synthesis and assembly of the virus genome. Glutamine is responsible for directly synthesizing nucleotides, which serve as the necessary building blocks for virus gene synthesis (Qiao et al., 2020). Our research has demonstrated comparable results on amino acid metabolism during MDV infection. The increased production of glutamate, valine, glycine, arginine, and proline were observed. These results were further confirmed by q-PCR data, which revealed a rise in the mRNA levels of enzymes involved in amino acid metabolism during MDV infection. The increased availability of amino acids during MDV infection could play a pivotal role in the rapid reproduction of the virus, by providing the building blocks for viral protein synthesis and particle assembly, though, GAD2 and ASS1 expression remained largely unchanged, while GOT1 expression first increased and then decreased. We speculated that these genes might not be necessary for MDV replication. Host cell metabolism provides all the energy and macromolecules needed for virus replication. Regulating host cell metabolism can change virus replication (Thai et al., 2015; Sanchez et al., 2017). Metabolic analysis of Newcastle disease virus (NDV) infection *in vivo* and *in vitro* have also revealed a fascinating correlation between the increased availability of amino acids and nucleotides and the replication of the virus (Liu et al., 2019). They found the enhanced production of viral proteins and the amplification of the NDV genome can be attributed to this increase in the metabolic pool. Amino acids are essential components of all living organisms. Not only do they serve as the building blocks for the synthesis of proteins and other essential biological molecules, they can also be utilized as intermediate metabolites for the TCA cycle and gluconeogenesis. This highlights the multifaceted role of amino acids in supporting vital cellular processes. Recent metabonomic studies have uncovered intriguing evidence that viral infections can significantly alter amino acid metabolism. These findings provide novel insights into the intricate relationship between viral infections and cellular metabolism (Birungi et al., 2010; Fontaine et al., 2014; Singh et al., 2018), providing a new perspective on the intricate relationship between MDV and host metabolism and offer a deeper understanding of the mechanisms of MDV infection (Figure 6).

The TCA cycle and amino acid metabolism are essential for the viral replication. These metabolic pathways serve as essential sources of energy and building blocks for the virus, allowing for rapid replication (Ritter et al., 2010; Vastag et al., 2011; Sanchez and Lagunoff, 2015). In the course of viral replication, the TCA cycle has been identified as a significant factor in the life cycle of certain viruses, including human cytomegalovirus (HCMV). The conversion of glutamine into α-ketoglutaric acid, a process facilitated by the enzymes glutaminase and glutamate dehydrogenase, is necessary for HCMV replication. The TCA cycle then steps in to convert α-ketoglutarate into ATP, providing the energy necessary for viral production. In fact, restoring ATP synthesis and viral production in glutamine-starved cells can be achieved by adding TCA cycle intermediates like α-ketoglutarate, oxaloacetic acid, or pyruvate, further emphasizing the TCA cycle’s involvement in HCMV replication (Chambers et al., 2010). Not all viruses, however, rely on the TCA cycle for replication. The proliferation of infectious splenic and renal necrosis virus does not require the TCA cycle to carry out its life cycle (Guo et al., 2019). In contrast, the results of a metabonomic analysis following MDV infection showed a significant increase in citrate and 2-oxoglutarate levels at 24 and 36 hpi, which suggests a potential role of the TCA cycle in MDV replication. These findings indicate that the TCA cycle is involved in the replication of a variety of viruses, highlighting its importance in the field of virology.

The metabolic network of MDV remains a largely unexplored sphere of the virus-host relationship. Boodhoo et al. (2019) used gas chromatography-mass spectrometry (GC-MS) analysis to study MDV-infected CEFs and found that *de novo* fatty acid production and prostaglandin E2 synthesis were essential for MDV replication. Additionally, they observed that the virus triggered glycolysis and glutaminolysis to generate energy and metabolites necessary for its proliferation (Boodhoo et al., 2020b). However, slight differences in our findings from those of the previous study are due to differences in the test methodologies used (GC-MS vs. LC-MS) and the time of infection. Despite these differences, the results highlight the complex interplay between the virus and host metabolism in the replication of MDV. The replication process of MDV needs to accurately regulate the metabolic pathway of host cells, so as to provide sufficient energy and biomacromolecule building blocks for itself. Glycolysis is one of the main ways of cell energy production. It breaks down sugar molecules to produce ATP, which provides power for various activities of cells. However, MDV takes this process as a strategy, using the energy generated by glycolysis to provide energy for its own replication and assembly. At the same time, the replication of MDV also depends on the synthesis of amino acids. Amino acids are the basic units of proteins, and proteins are essential for virus replication and assembly. MDV controls the amino acid synthesis of host cells to meet most of the amino acids needed for its own replication. These amino acids are not only used for the synthesis of viral proteins, but also important raw materials for the synthesis of viral genetic material DNA and RNA. Our research and Boodhoo’s research jointly reveal how MDV cleverly uses and reshapes the metabolic network of host cells to facilitate its own replication and assembly. Further research in this area will help us better understand the mechanisms underlying viral replication and pave the way for new therapeutic approaches.

Based on an untargeted LC-MS metabolomic profile, this study demonstrates that MDV manipulates the metabolic landscape of CEFs by inducing alterations in various metabolic pathways, including purine metabolism, pyrimidine metabolism, amino acid metabolism, and the TCA cycle. These modifications in host cell metabolism supply the essential energy and substrate resources for successful virus replication. These findings open up new avenues for exploring the treatment and control of MD. By observing the metabolic changes after MDV infection, it is helpful to understand the mechanism of MDV replication and transmission in host cells. In particular, we have gained a new understanding of the use and disruption of MDV in the metabolism of amino acids, energy and nucleic acids in host cells. These findings may help us design new antiviral strategies. In addition, understanding how MDV affects the metabolic process of poultry can help us optimize the feeding and management of poultry to reduce the transmission and infection of MDV.

## Data availability statement

The original contributions presented in the study are included in the article/Supplementary material, further inquiries can be directed to the corresponding author.

## Author contributions

This manuscript was written by QW and AQ. Experiment and data analysis were performed by QW and BS. The study was designed by QW, AQ, GY, XZ, HS, KQ, and JY. All authors contributed to the article and approved the submitted version.
