# Supplementary figures and images for: Metabolomic profiling of Marek’s disease virus infection in host cell based on untargeted LC-MS
Source: Front Microbiol. 2023 Nov 9;14:1270762. doi: 10.3389/fmicb.2023.1270762 (PMC10666056; doi:10.3389/fmicb.2023.1270762)

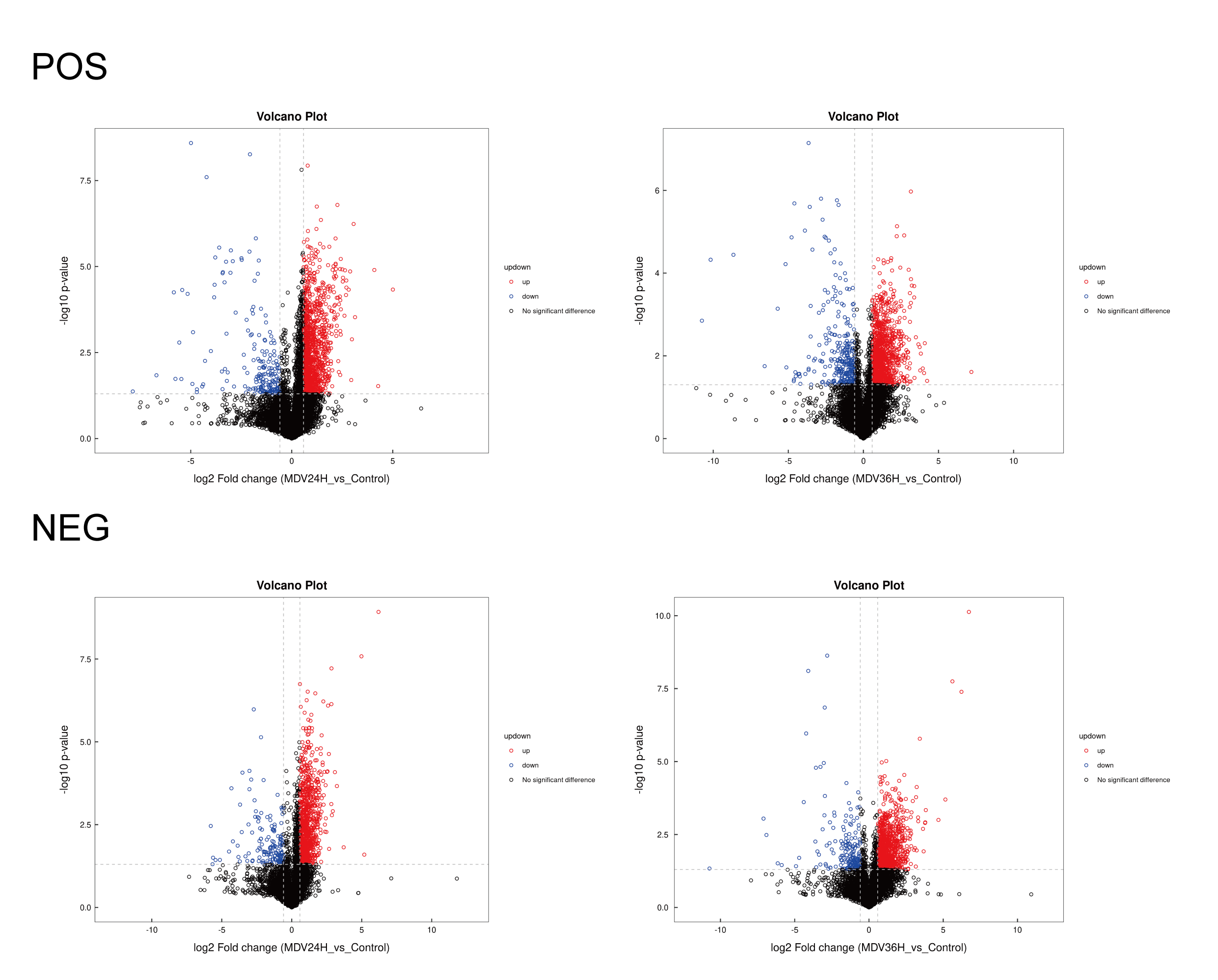

Supplement: Supplementary file 4 [file Image_1.TIF]
